# Susceptibility of different cell lines to the novel canine coronavirus CCoV‐HuPn‐2018

**DOI:** 10.1111/irv.12882

**Published:** 2021-07-01

**Authors:** Anfal Abdelgadir, Anastasia N. Vlasova, Gregory C. Gray

**Affiliations:** ^1^ Division of Infectious Diseases, School of Medicine Duke University Durham NC USA; ^2^ Duke Global Health Institute Duke University Durham NC USA; ^3^ Food Animal Health Research Program, Ohio Agricultural Research and Development Center, College of Food, Agricultural and Environmental Sciences, Department of Veterinary Preventive Medicine The Ohio State University Wooster OH USA; ^4^ Global Health Research Center Duke Kunshan University Kunshan China; ^5^ Program in Emerging Infectious Diseases Duke‐NUS Medical School Singapore

**Keywords:** alphacoronavirus, canine coronavirus, CCoV‐HuPn‐2018, cell lines, receptivity

Dear Editor,

1

Over the past few decades, we have witnessed the emergence of numerous novel viruses within the family Coronaviridae. These have included the swine acute diarrhea syndrome coronavirus (SADS‐CoV), the canine respiratory coronavirus (CRCoV), the feline coronavirus serotype II (FCoV‐II), and the latest severe acute respiratory syndrome coronavirus 2 (SARS‐CoV‐2).^1^, ^2^, ^3^, ^4^, ^5^ Coronaviruses have increased opportunities for mutation and spill‐over due to the frequent recombination and mutation events during replication, which helps them generate new viral threats. In fact, it is understood that all currently recognized human coronaviruses, HCoV‐229E, HCoV‐OC43, HCoV‐NL63, HCoV‐HKU1, SARS‐CoV, MERS‐CoV, and SARS‐CoV‐2, are zoonotic in origin.^6^, ^7^ However, evidence for canine and feline coronaviruses spilling over to humans has been sparse.

In a recent study evaluating a molecular diagnostic assay for coronaviruses, our team found evidence of canine coronavirus (CCoV) in eight patients hospitalized with pneumonia in Sarawak, Malaysia between 2017 and 2018.^8^ Further analysis and viral isolation were then conducted in canine fibroblast tumor cells (A72). Among the eight samples, one specimen yielded a viral isolate, which was characterized by complete genome sequencing. The identified virus was a novel canine–feline recombinant alphacoronavirus (genotype II) that was named CCoV‐HuPn‐2018.^9^

We sought to assess the receptivity of different animal and human cell lines to the novel canine coronavirus CCoV‐HuPn‐2018 in comparison to another canine coronavirus, CCoV‐UCD1 and a seasonal human coronavirus, HCoV‐229E. The studied cell lines included adenocarcinomic human alveolar basal epithelial cells (A549), the human lung fibroblast cell line (MRC‐5), Madin–Darby canine kidney (MDCK) cells, African green monkey kidney epithelial cells (VeroE6), pig testis cells (ST), and mink lung epithelial cells (Mv1Lu). A72 cells were used as a positive control for the CCoVs.

In 24‐well plates, monolayers of MDCK, ST, A549, MRC.5, and A72 cells were inoculated with the two canine coronaviruses, CCoV‐HuPn‐2018 and CCoV‐UCD1. CCoV‐HuPn‐2018 was also inoculated in Mv1Lu and VeroE6 cells. The human coronavirus HCoV‐229E was inoculated in monolayers of MDCK, ST, A549 and MRC.5 cells. Median tissue culture infectious dose (TCID50) was calculated for each virus using the Reed–Muench method,^10^ and inoculations were conducted at a multiplicity of infection (MOI) of 0.1. Cells were then incubated for 1 h at 37°C and 5% CO_2_, except A72 cells which were incubated without CO_2_. Following the incubation, virus was removed, and cells were washed once with phosphate‐buffered saline (PBS), then fresh infection media containing 2% fetal bovine serum was added. Cells were monitored for cytopathic effect (CPE) every 24 h. Cells and supernatant were harvested at 0‐, 40‐, 72‐, and 192‐h postinoculation. RNA was extracted using the QIAamp Viral RNA Mini Kit (QIAGEN, Inc., Valencia, CA) and screened with a real‐time reverse transcription polymerase chain reaction (qRT‐PCR) assay specific for the virus.^9^, ^11^ Virus culture was considered positive when the cycle threshold (Ct)^7^ value was at least 2 points below the 0‐h inoculum and CPE was present.

CPE was observed 40‐h postinoculation in A72 cells inoculated with CCoV‐HuPn‐2018 and CCoV‐UCD1 and confirmed with qRT‐PCR (Table 1). No increase in the viral replication was observed in MDCK, ST, A549, MRC.5, Mv1Lu, and VeroE6 cells even after 192‐h postinoculation, suggesting that these cell lines are not permissive for CCoV‐HuPn‐2018 and CCoV‐UCD1 (Figure S1).

CPE was observed in MRC5 cells inoculated with HCoV‐229E beginning at 72‐h postinoculation. This observation was also confirmed by qRT‐PCR as Ct values were significantly lower than the original result. MDCK, ST, and A549 cells were monitored up to 192‐h postinoculation, and no CPE was observed in these cells nor were positive qRT‐PCR results detected.

**TABLE 1:** Susceptibility of cells to CCoV‐HuPn‐2018 as assessed by cytopathic effect and qRT‐PCR

Cell line	Species	Cell type	CPE	Quantitative PCR Ct (hours 0, 72, 192)
A549	Human	Lung carcinoma epithelium	−	24.7, 27, 29.9
MRC‐5	Human	Fetal lung fibroblast	−	24.0, 30.7, 33.4
MDCK	Canine	Kidney epithelium	−	24.6, 29.6, 33.1
A72^a^	Canine	Tumor fibroblast	+	25.4, 17.7, 16.4
Vero E6	African green Monkey	Kidney epithelium	−	25.3, 28.7, 31.3
ST	Swine	Fetal testes	−	24.2, 28.5, 31.7
Mv1Lu	Mink	Lung epithelium	−	25.2, 28.1, 31.0

Abbreviations: CPE, cytopathic effect; Ct, cycle threshold; qRT‐PCR, quantitative reverse transcription polymerase chain reaction.

^a^ CPE was observed 40‐h postinoculation in this cell line.

The ability of the CCoVs to form CPE in A72 cells and the HCoV‐229E to infect MRC.5 cells has been previously described.^9^, ^12^, ^13^ Our experiments suggest that the studied human lung cells are not receptive for CCoV‐HuPn‐2018 infection and replication, despite their expression of APN receptors. However, previous studies have suggested that some coronaviruses are resistant to cell culture.^14^, ^15^ Additionally, permissiveness of various cell lines to coronavirus infection in vitro does not always recapitulate the in vivo tissue and host.^16^ In vitro infection of this novel CCoV in human cell lines is challenging and requires further understanding of the virus pathogenesis and infection initiation in the human respiratory system.

## CONFLICT OF INTEREST

None declared.

## AUTHOR CONTRIBUTIONS

**Gregory Gray:** Conceptualization; funding acquisition; supervision. **Anfal Abdelgadir:** Investigation. **Anastasia Vlasova:** Conceptualization; investigation; methodology; supervision.

## Supporting information


**Figure S1.** Microscopic images of uninfected cells (right column) and infected cells (left column) with the novel canine coronavirus CCoV‐HuPn‐2018.
**A.** A549 cells 72 hours post‐inoculation. **B.** MRC‐5 cells 72 hours post‐inoculation. **C.** MDCK cells 72 hours post‐inoculation. **D.** A72 cells 72 hours post‐inoculation. **E.** VeroE6 cells 72 hours post‐inoculation. **F.** ST cells 72 hours post‐inoculation. **G.** Mv1Lu 72 hours post‐inoculation.
