# Supplementary material for: Susceptibility of different cell lines to the novel canine coronavirus CCoV‐HuPn‐2018
Source: Influenza Other Respir Viruses. 2021 Jul 1;15(6):824–5. doi: 10.1111/irv.12882 (PMC8542962; doi:10.1111/irv.12882)
Supplement: Supplementary file 1 — Figure S1. Microscopic images of uninfected cells (right column) and infected cells (left column) with the novel canine coronavirus CCoV‐HuPn‐2018. A. A549 cells 72 hours post‐inoculation. B. MRC‐5 cells 72 hours post‐inoculation. C. MDCK cells 72 hours post‐inoculation. D. A72 cells 72 hours post‐inoculation. E. VeroE6 cells 72 hours post‐inoculation. F. ST cells 72 hours post‐inoculation. G. Mv1Lu 72 hours post‐inoculation. [file IRV-15-824-s001.docx]

**Legend for Supplemental Figure:**

Supplemental Figure 1. Microscopic images of uninfected cells (right column) and infected cells (left column) with the novel canine coronavirus CCoV-HuPn-2018.

**A.** A549 cells 72 hours post-inoculation. **B.** MRC-5 cells 72 hours post-inoculation. **C.** MDCK cells 72 hours post-inoculation. **D.** A72 cells 72 hours post-inoculation. **E.** VeroE6 cells 72 hours post-inoculation. **F.** ST cells 72 hours post-inoculation. **G.** Mv1Lu 72 hours post-inoculation.

*
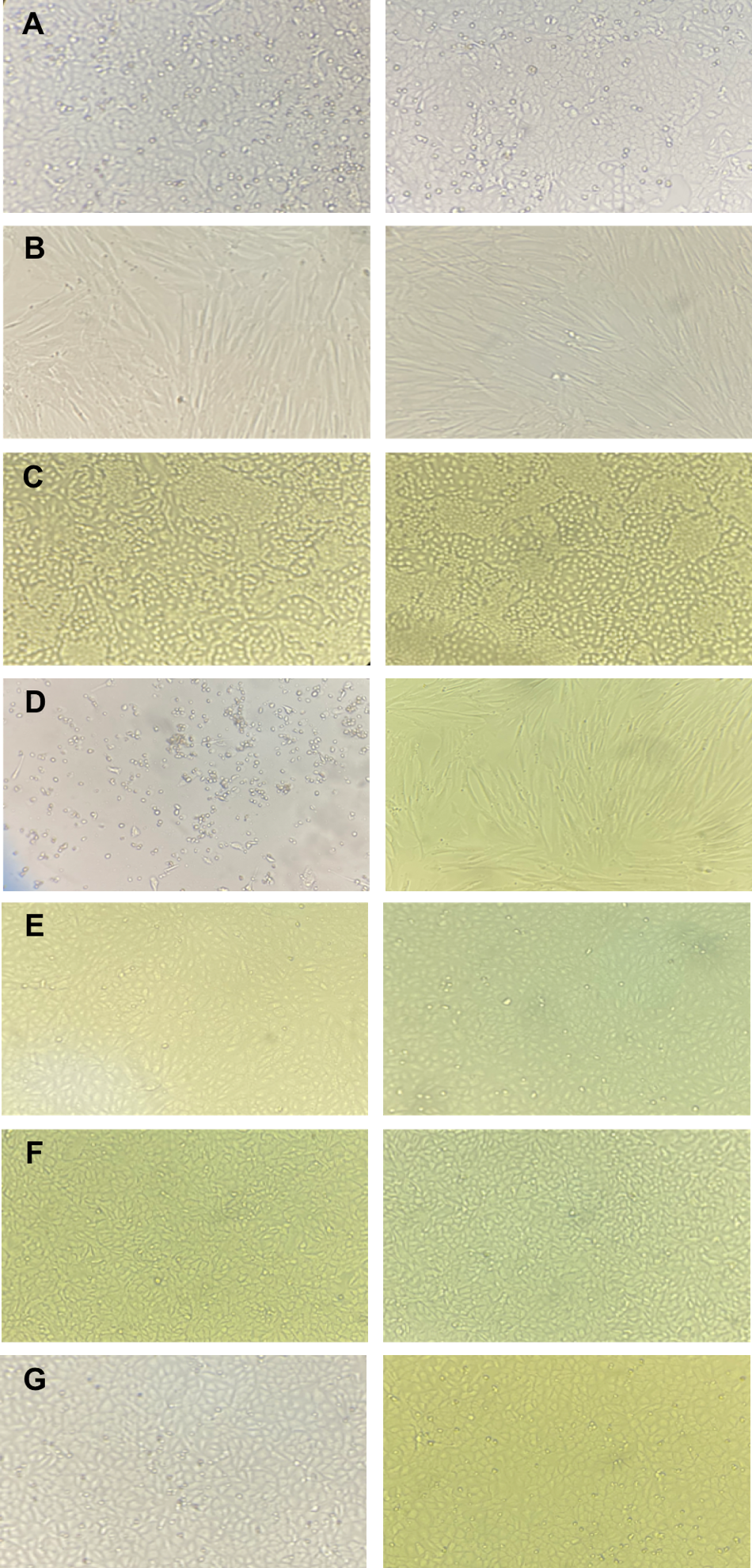
*
